# Glucocorticoid-Induced Autophagy in Osteocytes

**DOI:** 10.1002/jbmr.160

**Published:** 2010-06-18

**Authors:** Xuechun Xia, Rekha Kar, Jelica Gluhak-Heinrich, Wei Yao, Nancy E Lane, Lynda F Bonewald, Sondip K Biswas, Woo-Kuen Lo, Jean X Jiang

**Affiliations:** 1 Department of Biochemistry, University of Texas Health Science Center San Antonio, TX, USA; 2 Department of Orthodontics, University of Texas Health Science Center San Antonio, TX, USA; 3 Center for Healthy Aging, Internal Medicine, University of California at Davis Medical Center Sacramento, CA, USA; 4 Department of Oral Biology, School of Dentistry, University of Missouri Kansas City, MO, USA; 5 Department of Neurobiology, Morehouse School of Medicine Atlanta, GA, USA

**Keywords:** glucocorticoid, osteocyte, autophagy, viability

## Abstract

Glucocorticoid (GC) therapy is the most frequent cause of secondary osteoporosis. In this study we have demonstrated that GC treatment induced the development of autophagy, preserving osteocyte viability. GC treatment resulted in an increase in autophagy markers and the accumulation of autophagosome vacuoles in vitro and in vivo promoted the onset of the osteocyte autophagy, as determined by expression of autophagy markers in an animal model of GC-induced osteoporosis. An autophagy inhibitor reversed the protective effects of GCs. The effects of GCs on osteocytes were in contrast to tumor necrosis factor α (TNF-α), which induced apoptosis but not autophagy. Together this study reveals a novel mechanism for the effect of GC on osteocytes, shedding new insight into mechanisms responsible for bone loss in patients receiving GC therapy. © 2010 American Society for Bone and Mineral Research.

## Introduction

Glucocorticoids (GCs) are used extensively for the treatment of chronic inflammatory and autoimmune diseases. However, prolonged use of GCs results in reduction of bone mineral density (BMD), with fractures occurring in 30% to 50% of patients treated chronically with GCs.(1) Administration of GCs leads to decreased generation of osteoblasts and osteocytes, accompanied by a prolonged lifespan of osteoclasts.(2) Increased apoptosis of osteocytes and osteoblasts has been observed with high-dose GC treatment in mice and in clinical biopsy specimens in some, but not all, studies.(3–6) Apoptosis of osteocytes and osteoblasts could reduce bone formation and possibly weaken the bone structure, leading to an increase in fracture risk. However, neither the changes in bone metabolism and bone architecture nor the modest amount of apoptosis of osteoblasts and osteocytes can explain the elevated bone fragility observed in GC-treated patients.(7) We have reported increased osteocyte lacunar size with a loss of perilacunar mineral with GC treatment, suggesting that the osteocyte is metabolically stressed or compromised.(8) In this model of GC treatment, less than 5% of osteocytes were found to be apoptotic, suggesting another effect of GCs. Therefore, the focus of this study was to determine whether autophagy, a protective mechanism by which cells can respond to stress, may be relevant to GC-induced changes to osteocytes.

Autophagy is a lysosomal degradation pathway that is essential for cell growth, survival, differentiation, development, and homeostasis.(9) It is a tightly regulated process that helps to maintain a balance among the synthesis, degradation, and subsequent recycling of cellular products. During autophagy, parts of the cytoplasm and intracellular organelles are sequestered within autophagic vacuoles that are eventually delivered to lysosomes for bulk degradation.(10) Autophagy has been proposed as a “double-edged sword” in heart tissue.(11) Autophagy can protect the cells from apoptosis by removing oxidatively damaged organelles. On the other hand, excess autophagy can destroy cellular components. Autophagy therefore can preserve viability or, alternatively, can be a self-destructive process that leads to cell death.(12) However, whether autophagy is involved in the effect of GCs on bone cells was unknown previously.

In this study, we show that GC treatment of osteocytes and mice resulted in the development of autophagy. Inhibition of autophagy led to augmentation of the effect of GCs on cell viability. Our observations offer novel findings that autophagy is a major mechanism used by osteocytes in self-protection against the detrimental effect of GCs that results in bone loss.

## Materials and Methods

### Cell culture

MLO-Y4 cells were cultured on collagen-coated (rat tail collagen type I, 0.15 mg/mL) surfaces and were grown in phenol red–free α-MEM supplemented with 2.5% fetal bovine serum (FBS) and 2.5% bovine calf serum (BCS) and incubated in a 5% CO_2_ incubator at 37°C, as described previously.(13)

### Isolation of chicken primary osteocytes

Preparation of primary osteocytes from chicken calvarias was based on published procedures.(13) Briefly, calvarial bone was dissected from 16-day-old embryonic chicks. The soft tissues and osteoid were removed by collagenase, followed by decalcification using EDTA. The final particles were treated with collagenase and agitated vigorously to release osteocytes and cultured in α-MEM supplemented with 10% FBS for 1 hour at 37°C.

### Cell viability assay

MLO-Y4 cells were seeded in 96-well flat-bottomed plates and incubated overnight at 37°C. After exposure to dexamethasone (Dex) at various concentrations and times, the cells were exposed to 10 µL of WST-1 reagent for 1 to 2 hours at 37°C. The absorbance at 450 nm was measured using a microplate reader. Alternatively, MLO-Y4 cells were plated in a 6-well plate overnight at 37°C. After the treatment with Dex, the number of viable cells was counted. The annexin V staining of Dex or TNF-α/CHX-treated MLO-Y4 cells was performed using the Annexin-V-FLOUS Staining Kit (Roche Applied Science, Indianapolis, IN, USA). Propidium iodide (PI) staining also was performed at the same time using the same kit.

### LDH-cytotoxicity assay

A Lactate Dehydrogenase (LDH) Cytotoxicity Assay Kit (BioVision, Mountain View, CA, USA) was used for quantification of plasma membrane damage. MLO-Y4 cells were seeded in a 96-well plate overnight at 37°C. After treatment with Dex, the plates were centrifuged at 250*g* for 10 minutes. Then 100 µL per well of supernatant was transferred into corresponding wells of a clear 96-well plate and followed by addition of 100 µL of the reaction mixture to each well and incubation for 30 minutes in the dark. The absorbance at 490 nm was measured using a microplate reader. The maximum release of LDH was determined by incubation of the cells in 1% Triton X-100 for 1 hour in culture medium. The percentage of cytotoxicity was determined by the following equation: cytotoxicity (%) = [(test samples – blank)/(maximum – blank)] × 100.

### DAPI staining for detection of chromatin condensation

MLO-Y4 cells were treated with Dex, fixed in 4% paraformaldehyde for 30 minutes, incubated with DAPI (0.2 µg/mL) for 10 minutes, and examined by fluorescence microscopy.

### Mitochondrial membrane potential

After treatment with Dex, cells were collected by trypsinization, stained with 100 nM TMRE in phenol red–free α-MEM medium without serum for 20 minutes at 37°C, and then analyzed with flow cytometry.

### Detection and quantification of acidic vesicular organelles with acridine orange staining

MLO-Y4 cells were stained with acridine orange (1 µg/mL) for 15 minutes at 37°C. For quantification of the development of acidic vesicular organelles, the cells were stained with acridine orange (1 µg/mL) for 15 minutes at 37°C and measured by flow cytometry (gate set at 5%; FACScan Flow Cytometer, BD Biosciences, San Jose, CA, USA) at green (510 to 530 nm) and red (>650 nm) fluorescence emission from 2 × 10^4^ cells illuminated with blue (488 nm) excitation light. The data were analyzed by BD CellQuest software (San Jose, CA, USA).

### Detection of autophagic vacuoles with monodansylcadaverine (MDC)

Cells were incubated with MDC at a concentration of 0.05 mM in PBS at 37°C for 10 minutes. Cells then were washed four times with PBS and analyzed by fluorescent microscopy (excitation 380 nm, emission 525 nm). Alternatively, cells were collected in 10 mM Tris-HCl, pH 8.0, containing 0.1% Triton X-100, and the supernatant was assayed on a Fluoro-Max-3 fluorometer (Horiba Jobin Yvon, Edison, NJ, USA) (excitation 380 nm, emission 519 nm), and the intensity of the fluorescence was quantified.

### GFP-LC3 dots assay

A GFP-LC3 dots assay was performed as described previously.(14) Cells were transiently transfected with the GFP-LC3 vector. After overnight culture, the cells were treated with Dex, fixed with 4% paraformaldehyde, and examined under a fluorescence microscope. To quantify autophagic cells after Dex treatment, cells exhibiting GFP-LC3 punctuate dots were counted.

### Histologic sections and immunocytochemistry

Six-month-old male Swiss-Webster male mice were obtained from Charles River, Inc. (Wilmington, MA, USA) The mice were maintained on commercial rodent chow (22/5 Rodent Diet, Teklad, Madison, WI, USA) available ad libitum with 0.95% calcium and 0.67% phosphate. Mice were housed in a room that was maintained at 21°C with a 12-hour light/dark cycle. Slow-release pellets (Innovative Research of America, Sarasota, FL, USA) of placebo or 5 mg/60 day slow-release prednisolone pellets (group 2, *n* = 15) were administrated by subcutaneous implantation. The third lumbar vertebral body (LVB) were decalcified in 10% EDTA for 2 weeks and embedded in paraffin. Then 4-µm sections were collected using a Leica 2265 Microtome (Bannockburn, IL, USA). After deparaffinization and rehydration, sections were blocked in PBS containing 1% goat serum at room temperature for 1 hour and then labeled with 1:100 dilution of anti-LC-3 antibody for 1 hour and followed by incubation with the ABC reagent (Vector, Burlingame, CA, USA) at room temperature for 30 minutes. Alkaline phosphatase substrate solution was used to visualize immunoreaction sites.

### Gene microarray analysis

RNA was extracted from the long bones of placebo- or GC-treated mice. Purified total RNA (10 µg) from each animal (*n* = 3 to 5 per time point) was used for cDNA synthesis, which served as a template for in vitro transcription with biotin incorporation. All the microarray analyses were run for individual animals (*n* = 3 to 5 per group per time point) from placebo- or GC-treated groups euthanized on days 0, 7, 28, and 56. Fragmented biotinylated transcripts were hybridized to the Mouse Genome 430 2.0 Array (Affymetrix, Santa Clara, CA, USA) according to the manufacturer's protocol. Washing and staining of the arrays were performed on a Fluidics Station 450 (Affymetrix), and the arrays then were scanned using the GeneChip Scanner 3000 (Affymetrix). Raw intensity data for 45,101 probe sets were processed and obtained from the scanned image files by the GeneChip Operating Software (GCOS, Affymetrix).

## Results

### Reduction of MLO-Y4 cell number by Dex

**Fig. 1:**
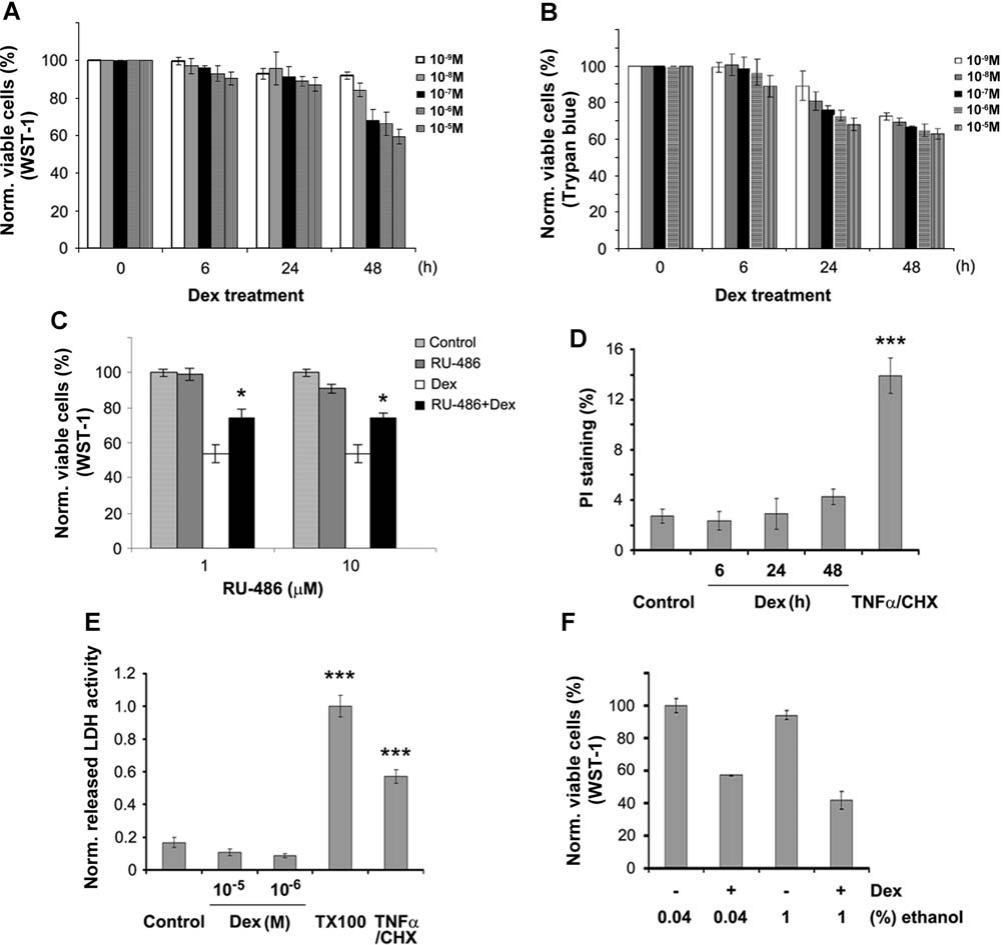
Reduction of MLO-Y4 cell number by Dex. (*A*, *B*) MLO-Y4 cells were treated with Dex at 10^−5^, 10^−6^, 10^−7^, 10^−8^, and 10^−9^ M for 0, 6, 24, and 48 hours. The number of viable cells was measured using WST-1 or trypan blue dye exclusion assay. No or few trypan blue cells were detected. (*C*) Cells were pretreated with 1 or 10 µM RU486 prior to the application of Dex, and the cell number was determined using WST-1. RU486 partially reversed the negative effects of Dex on the number of live MLO-Y4 cells. (1 or 10 µM RU486 plus Dex–treated versus only Dex-treated samples: ^*^p < .05.) (*D*) Cells were treated with 10^−6^ M Dex for 6, 24, and 48 hours or with 10 ng/mL of TNF-α/CHX for 6 hours. Cell number was analyzed by PI staining, and the number of PI-labeled cells was quantified. (TNF-α/CHX-treated versus control samples: ^***^p < .001.) (*E*) Cell membrane damage was measured by release of the cytoplasmic enzyme lactate dehydrogenase (LDH) from MLO-Y4 cells treated with Dex or TNF-α/CHX. (48 hours of Dex- or TNF-α/CHX-treated versus control: ^***^p < .001.) (*F*) Cells were treated with 0.04% or 1% ethanol or Dex dissolved in 0.04% or 1% ethanol, and cytotoxicity was determined using WST-1 assay. All data are presented as mean ± SD and *n* = 3.

To determine the effect of GCs on the number of osteocytic MLO-Y4 cells, the cells were treated with various concentrations of Dex (10^−9^ to 10^−5^ M) or ethanol alone for 6, 24, or 48 hours, and the normal cell number was determined using WST-1 and dead cells by trypan blue exclusion assays. Dex reduced the normal numbers of MLO-Y4 cells in a time- and dose-dependent manner compared with 0 time and no treatment (Fig. 1*A, B*). Surprisingly, very few dead cells were detected at 24 and 48 hours using trypan blue. We then evaluated the effect of RU486, a GC receptor antagonist, to study the mechanistic action of GCs.(15,16) RU486 by itself had no effect, but it significantly prevented the effect of Dex on reducing the numbers of normal cells (Fig. 1*C*). Next, PI staining and flow cytometric quantification were performed to examine cell death (Fig. 1*D*). Interestingly, there was a slight, but not significant, increase in PI staining after Dex treatment for 6, 24, and 48 hours (approximately 2% PI staining after 48 hours of Dex treatment), suggesting that very little cell death was occurring. In contrast, TNF-α/CHX treatment, reagents reported to cause apoptosis of MLO-Y4 cells,(17) lead to a dramatic increase in PI staining and cell death. To assess the extent of plasma membrane damage, the release of the cytoplasmic enzyme LDH was quantified after Dex treatment at 10^−6^ M or 10^−5^ M for 48 hours (Fig. 1*E*). This result shows that there was no increase of LDH release from MLO-Y4 cells treated with Dex. It has been reported that an insoluble hydrophobic form of Dex in aqueous solution is more toxic for cells.(18) To control for this potential side effect, we treated the cells with Dex in the presence of 0.04% or 1% ethanol (Fig. 1*F*). Ethanol did not exhibit any discernible effect on the number of MLO-Y4 cells. Together these results suggest that Dex treatment resulted in either a decrease in normal cell number or an inhibition of proliferation of MLO-Y4 osteocytes without apparent plasma membrane damage.

### Dex induced minimal MLO-Y4 cell apoptosis

**Fig. 2:**
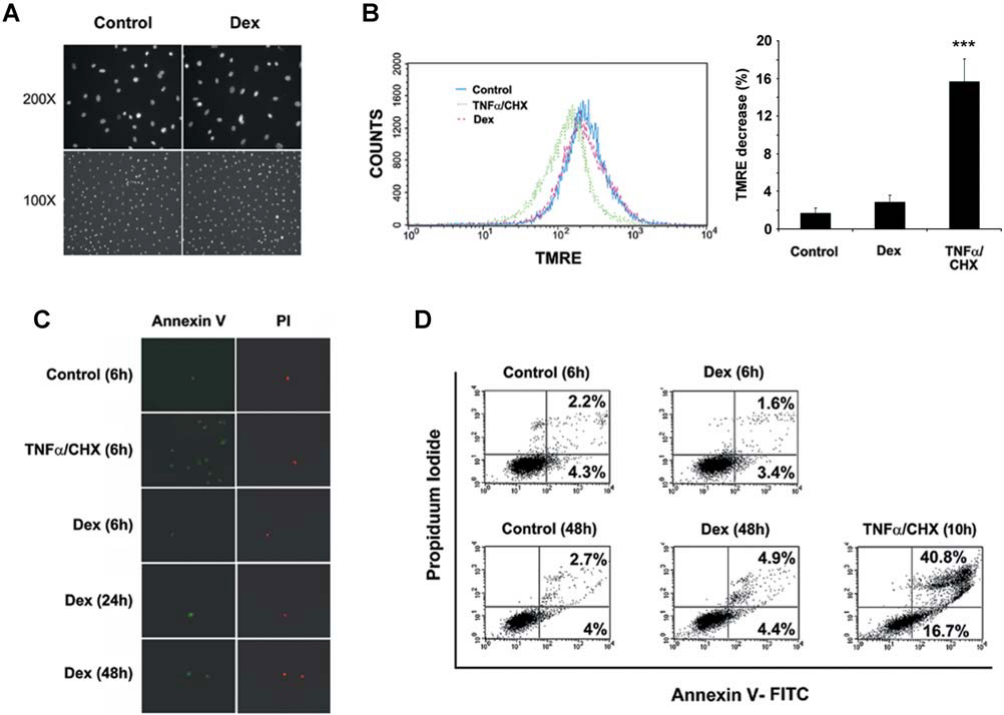
The major effects of Dex on MLO-Y4 cell number is not due to induced cell apoptosis. (*A*) MLO-Y4 cells were treated in the absence or presence of 10^−6^ M Dex for 48 hours and then labeled with DAPI. (*B*) Cells were treated with 10^−6^ M Dex or TNF-α/CHX for 48 hours, and suspended cells were stained with 100 nM TMRE and analyzed using flow cytometry and quantified (*right panel*). (TNF-α/CHX versus control and Dex-treated: ^***^p < .001.) (*C*) MLO-Y4 cells treated in the absence or presence of 10^−6^ M Dex for 6, 24, and 48 hours or TNF-α/CHX for 10 hours, labeled with Annexin-V-FLOUS Staining Kit and PI. (*D*) After treatment with 10^−6^ M Dex for 6 and 48 hours or TNF-α/CHX for 10 hours, detached and attached cells were analyzed using flow cytometry.

The preceding experiment indicated that apopotosis-related cell death may not be responsible for the reduced cell number. Therefore, we conducted several conventional assays to test for apoptosis. MLO-Y4 cells were treated with Dex at 10^−6^ M for 24 hours and then labeled with the nuclear marker DAPI (Fig. 2*A*). No significant difference in nuclear staining between control and Dex-treated cells was observed. The cell viability also was assessed by measuring the inner mitochondrial membrane potential by TMRE uptake (Fig. 2*B*). Compared with vehicle-treated controls (Fig. 2*B*, *left panel*, *blue*), TNF-α/CHX treatment resulted in a significant shift to the left of the peak (*green*) in the histogram, implying loss of mitochondrial membrane potential. Conversely, Dex treatment for 48 hours (*red*) did not result in a peak shift, suggesting that mitochondrial membrane potential was not altered by Dex. Quantitative assessment revealed a significant decrease in TMRE uptake by TNF-α/CHX but not by Dex treatment (Fig. 2*B*, *right panel*). Annexin V/PI staining is a rapid and sensitive method for the detection of apoptosis.(19) The cells stained with annexin V represent two types of cell responses. One is for cells in early apoptosis, which cannot be stained with PI, showing that the cell membrane is not “leaking.” These cells are in the lower right quadrant (Fig. 2*D*). When cells stain for both annexin V and PI, as shown in the upper right quadrant, this suggests that the cell membrane is “leaky” or that the cell is dead (Fig. 2*D*). TNF-α/CHX treatment for 10 hours significantly increased annexin V, but no significant increase was detected after Dex treatment for 6, 24, or 48 hours (Fig. 2*C*). Quantification using fluorescence-activated cell sorting (FACS) separation showed that TNF-α/CHX treatment for 10 hours significantly increased the number of annexin V^+^ as well as PI-labeled cells (two right quadrants, 57.5% versus 6.5% for control), but there was no increase in annexin V/PI labeling with Dex treatment for 6 or 48 hours (Fig. 2*D*; 6 hours: 5% versus 6.7% control; 48 hours: 9.3% versus 6.7%). Together these data show that unlike TNF-α/CHX, Dex treatment did not induce significant levels of apoptosis-related cell death in MLO-Y4 cells.

### Dex induces autophagy in MLO-Y4 cells

**Fig. 3:**
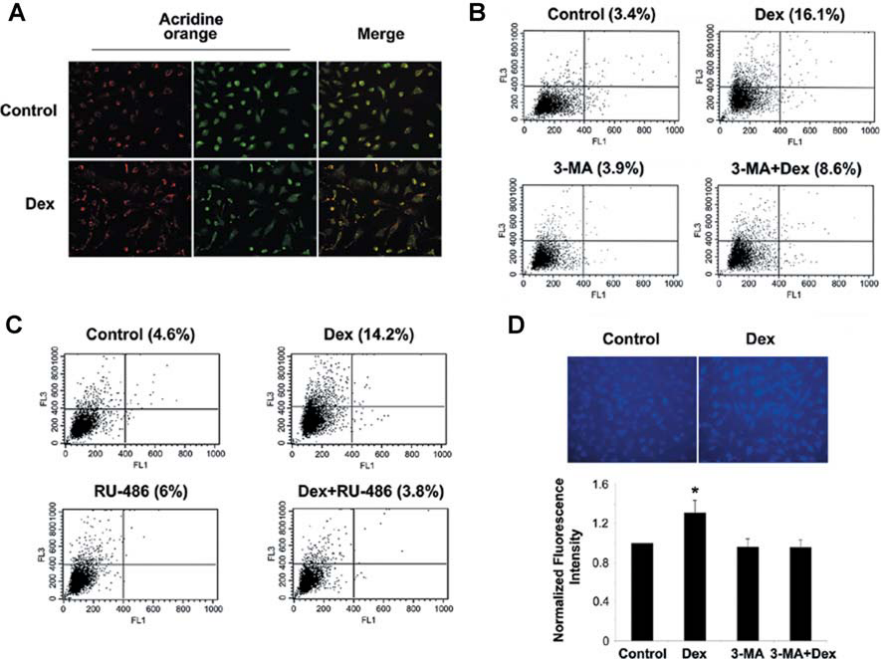
Induction of autophagy in MLO-Y4 cells by Dex. (*A*) Dex induced the development of autophagy. MLO-Y4 cells were treated in the absence or presence of 10^−6^ M Dex for 48 hours and labeled with acridine orange. (*B*) Cells were treated with 10^−6^ M Dex for 48 hours in the absence or presence of 0.5 mM 3-MA. 3-MA was added 1 hour prior to Dex treatment. Cells in suspension were labeled with acridine orange and quantified using flow cytometry (gate set at 5%), and the data were analyzed by CellQuest software. FL1-H indicates green color intensity (cytoplasm and nucleus), whereas FL3-H shows red color intensity (AVO). (*C*) Cells were treated with 10^−6^ M Dex for 48 hours in the absence or presence of 10 µM RU486. The latter was added 1 hour prior to Dex. Acridine orange–labeled cells in suspension were quantified using flow cytometry. (*D*) Cells were treated with 10^−6^ M Dex for 48 hours in the absence or presence of 0.5 mM 3-MA. 3-MA was added 1 hour prior to Dex treatment. Cells then were labeled with MDC, and the intensity of the staining was quantified by measuring fluorescence in cell lysate using a fluorometer. (Dex-treated versus others: ^*^p < .05.) The data are presented as mean ± SD and *n* = 3.

Autophagy is characterized morphologically by the accumulation of acidic vesicular organelles.(20) To determine if acidic vesicular organelles occur in Dex-treated MLO-Y4 cells, acridine orange was used. Staining of MLO-Y4 cells with acridine orange revealed the appearance of acidic vesicular organelles (AVOs) after Dex treatment, as visualized by a concentrated dye staining pattern in the cytoplasmic vacuoles with bright red fluorescence (Fig. 3*A*). Green fluorescence indicated nonacidic granules and remained relatively constant. Flow cytometric analysis was conducted to quantify acridine orange–labeled cells by using the FL3 channel to evaluate the bright red fluorescence and the FL1 channel for green fluorescence (Fig. 3*B*). Dex treatment induced a significant increase in the intensity of bright red fluorescence from 3.4% to 16.1%. 3-MA, an inhibitor of class III phosphatidylinositol-3 kinase (PI3K), which is known to inhibit autophagic sequestration,(21,22) partially suppressed the induction of autophagic vacuoles in Dex-treated cells (Fig. 3*B*). Pretreatment with RU486 completely reversed the stimulatory effect of Dex on autophagy, as shown by flow cytometry (Fig. 3*C*). These results suggest that the Dex-induced increase in bright red fluorescence is attributable to the development of AVOs associated with autophagy.

The presence of MDC has been used as a specific marker for the detection of autophagic vacuoles.(23) Dex significantly increased the accumulation of MDC compared with nontreated control cells, and this increase was completely reversed by 3-MA (Fig. 3*D*). These data suggest that Dex induces the formation of mature autophagic vacuoles.

**Fig. 4:**
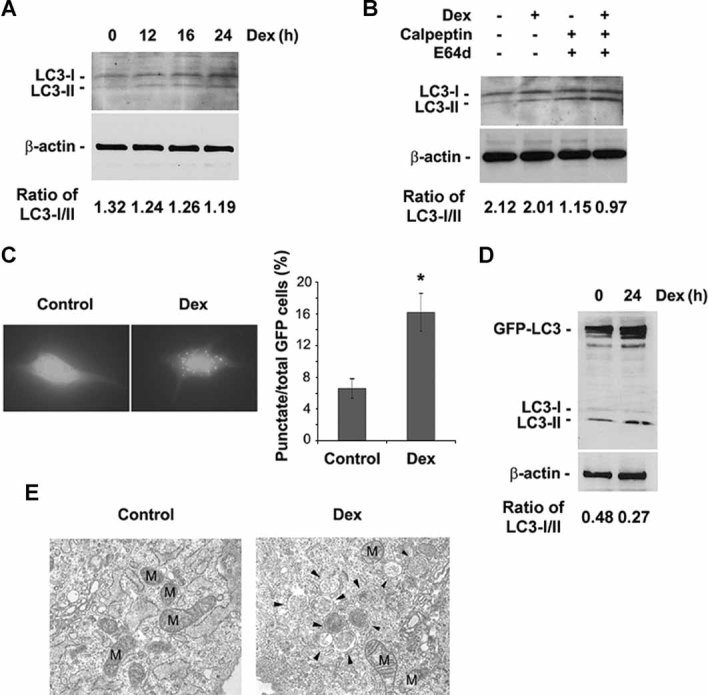
Dex increased LC3 levels and autophagosome development. (*A*) MLO-Y4 cells were treated with or without 10^−6^ M Dex for the indicated times and then subjected to immunoblotting analysis using anti-LC3 or anti-β-actin antibody. The band intensity was quantified, and the ratio of LC3-I/LC3-II was presented at the bottom of the blot. (*B*) Cells were treated with or without 10^−6^ M Dex for 24 hours in the absence or presence of E64d (10 mg/mL) or calpeptin (10 mg/mL). The latter two reagents were added 1 hour prior to Dex. The band intensity was quantified, and the ratio of LC3-I/LC3-II was presented at the bottom of the blot. (*C*) Cells were transiently transfected with GFP-LC3 construct for 24 hours, then treated with or without 10^−6^ M Dex for an additional 24 hours, and then examined by fluorescence microscopy. The percentage of GFP-LC3^+^ cells with GFP-LC3 punctate dots was quantified by counting the number of cells showing the punctate pattern of LC3-GFP in 100 GFP^+^ cells. (Dex versus control: ^*^p < .05.) The data are presented as mean ± SD and *n* = 3. (*D*) Cells were treated with 10^−6^ M Dex for 24 hours after being transiently transfected with GFP-LC3 construct for 24 hours. Cell lysates were analyzed by Western blot. The band intensity was quantified, and the ratio of LC3-I/LC3-II was presented at the bottom of the blot. (*E*) Cells were treated with 10^−6^ M Dex for 24 hours, and fixed cells were processed for thin-section electron microscopy. M indicates mitochondrial structures, and arrowheads indicate autophagosomes.

The expression of microtubule-associated protein light chain 3 (LC3) and the increased level of LC-3-II are important markers for autophagy development.(21) Consistently, a time-dependent decrease (after 12, 16 and 24 hours of Dex treatment) of the ratio of LC3-I to LC3-II was observed in MLO-Y4 cells treated with Dex (Fig. 4*A*). The further accumulation of LC3, especially LC3-II, was observed when cells were treated with the lysosomal inhibitors E64d and calpeptin (Fig. 4*B*). This result indicates that Dex-induced upregulation of autophagy is facilitated primarily through biosynthesis but not through the blockage of LC3-II degradation.(24)

GFP-tagged LC3 has been used as a sensitive method to demonstrate the induction of autophagy.(21,25,26) MLO-Y4 cells that expressed GFP-LC3 showed a diffuse distribution of green fluorescence in the absence of Dex. In contrast, treatment with Dex increased the number of “dots” or a punctate pattern and also fluorescence intensity (Fig. 4*C*, *left panel*), indicating that LC3 was recruited and aggregated on the membrane during Dex-induced autophagy. Quantification of these data suggests that a significantly increased number of Dex-treated cells exhibited the aggregation of LC3 compared with untreated cells (approximately 16% versus 6%). Western blot also showed the increase in GFP-LC3-II level by Dex treatment (Fig. 4*D*). Thin-section electron microscopic analysis showed the accumulation of membranous vesicles with cellular contents (*solid arrowheads*) in Dex-treated cells, but such structures were barely detectable in vehicle-treated control cells (Fig. 4*E*). Collectively, these results suggest that Dex-induced autophagy in MLO-Y4 cells was associated with induction as well as processing of LC3-I to LC3-II involved in the formation of autophagosomes.

**Fig. 5:**
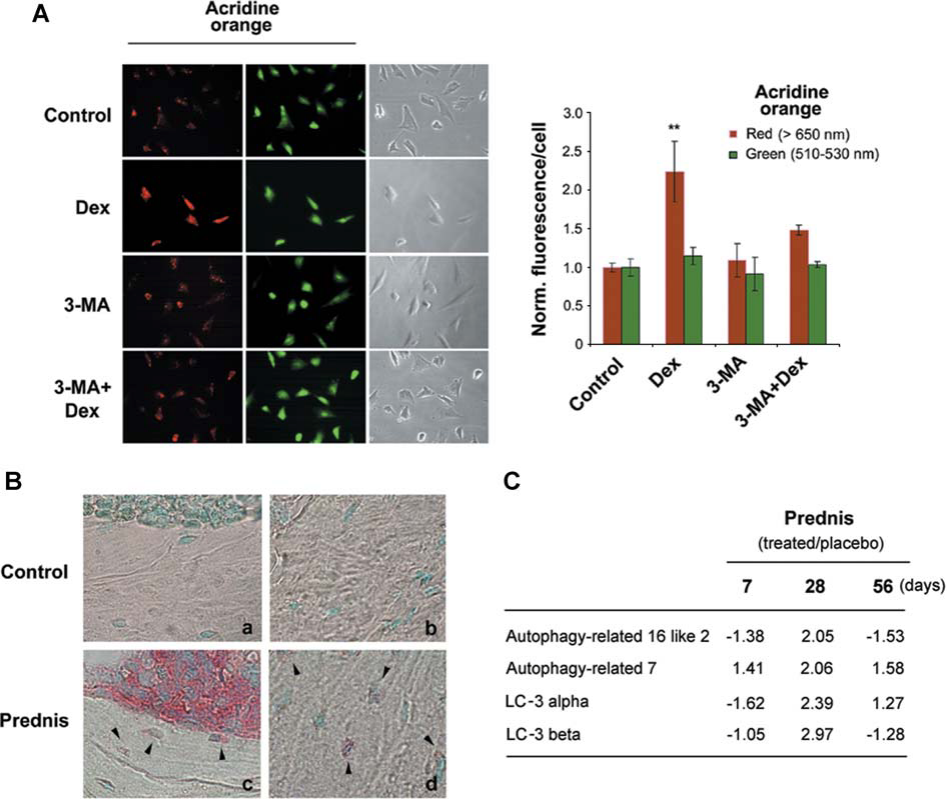
GCs increased markers of autophagy in primary osteocytes and in osteocytes within bone tissues from GC-treated mice. (*A*) Primary osteocytes isolated from chicken calvaria were treated with or without 10^−6^ Dex for 48 hours in the absence or presence of 3-MA (0.5 mM). 3-MA was added 1 hour before Dex treatment. Cells then were labeled with acridine orange, and green and red fluorescence was detected in acridine orange–stained cells using a fluorescence microscope (*left panel*). The staining intensity of acridine orange in the acquired images was quantified with NIH ImageJ analysis software (*right panel*). About 100 cells per sample were analyzed and normalized to control. (Red fluorescence in Dex-treated cells versus other treatment: ^**^p < .01; ^*^p < 0.05.) The data are presented as mean ± SD and *n* = 3. (*B*) Mice were treated with 5 mg of prednisolone for 60 days (prednis) (*c* and *d*) or with placebo (controls) (*a* and *b*). Paraffin sections were immunolabeled with anti-LC3 antibody, followed by incubation with AP-linked anti-rabbit secondary antibody, and counterstained with methyl-green. Arrowheads indicate LC3-labeled osteocytes. (*C*) RNA was extracted from the long bones of placebo- or prednisolone (prednis)–treated mice, gene microarray was conducted, and raw data were processed. Transcripts related to autophagy are increased after prednisolone treatment.

To further validate the development of autophagy in osteocytes, we used primary osteocytes isolated from chicken calvaria (Fig. 5*A*). Similar to our observation in MLO-Y4 cells, Dex treatment significantly increased autophagy, indicated by red fluorescence with acridine orange staining (Fig. 5*A*, *right panel*), and this increase was significantly inhibited by 3-MA (Fig. 5*A*, *right panel*).

The development of autophagy also was observed in osteocytes in mice treated with prednisolone (5 mg, 60-day slow-release pellet) for 2 months (Fig. 5*B*). LC3^+^ cells (red-brown staining, *arrowheads*) were visible in osteocytes of prednisolone-treated (Fig. 5*B*, panels *c* and *d*) but not in those of control mice (panels *a* and *b*), suggesting that autophagy by GCs occurs in osteocytes in vivo. Using gene microarray analysis, we found that the messenger levels of several autophagy markers, including LC-3α, LC-3β, autophagy-related 16 like 2, and autophagy-related 7, were elevated in bone samples of mice under chronic prednisolone treatment (Fig. 5*C*). Remarkably, compared with placebo (PL) control, the most dramatic increase occurs after 28 days of the GC treatment, which may indicate that a self-preservation mechanism was active during this time period.

### Inhibition of autophagy leads to a further reduction of normal cell numbers in response to Dex

**Fig. 6:**
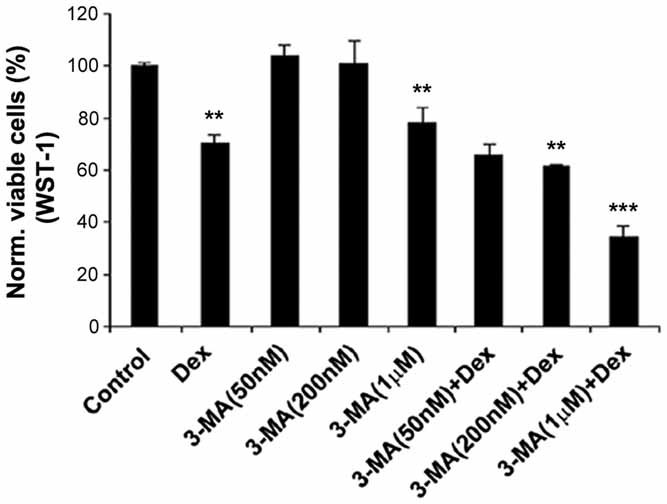
The reduction in normal cell number by Dex was augmented by 3-MA. MLO-Y4 cells were pretreated in the absence or presence of 50 and 200 nM and 1 µM of 3-MA prior to application of 10^−6^ M Dex. Cell viability was determined using WST-1. [Dex or 3-MA (1 µM) versus control: ^**^p < .01; 3-MA (200nM) + Dex versus Dex: ^**^p < .01; 3-MA (1 µM) + Dex versus Dex: ^***^p < .001.] All data are presented as mean ± SD and *n* = 3.

To determine whether autophagy is involved with the effects of Dex on osteocytes, we cotreated MLO-Y4 cells with Dex and 3-MA (Fig. 6). Increasing concentrations of 3-MA resulted in reduced numbers of cells measured by WST-1, suggesting that autophagy plays a role in maintaining cell survival under control conditions. More important, the reduction in the number of cells by Dex was significantly augmented by 3-MA at 200 nM and 1 µM, suggesting that autophagy plays a critical role in protecting the cells from the effects of Dex.

## Discussion

GCs are effective anti-inflammatory and autoimmune modulating agents, but GC treatment alters bone metabolism and increases bone fragility and subsequent bone fractures. We have shown that autophagy could be a major mechanism by which the osteocyte responds to elevated GCs. GC treatment resulted in a significant increase in markers of autophagy both in vitro and in vivo. GC treatment decreased the number of MLO-Y4 osteocytes in vitro, but no significant cell death was observed. Moreover, when autophagy was inhibited, a further decrease in metabolically normal cell number was observed with GC treatment. Together, our study suggests that autophagy is a likely self-protective process in osteocytes in response to the insult of GCs.

A previous study reported that GCs induce apoptosis of osteocytes, reporting nearly 15% to 20% apoptotic cells in GC-treated mice.(3) Interestingly, Lane and colleagues(8) reported increased osteocyte lacunar size and loss of mineral around the osteocytes, suggesting that a metabolic change had occurred in the presence of GC treatment. Very few apoptotic cells were observed. This led us to determine whether another mechanism(s) such as autophagy could be occurring in osteocytes that may account for the localized osteocyte perilacunar changes observed with GC treatment. By using multiple cell viability assays, we found that Dex reduced the number of osteocytes by over 50%. However, we did not detect significant levels of cell death, as determined by trypan blue and PI staining, consistent with other GC studies.(8,27) It has been postulated that GC receptor binding to mitochondrial membranes may regulate membrane potential and integrity. However, using TMRE dye, we failed to observe any alteration in the mitochondrial membrane, indicating that mitochondrial dysfunction was not induced by GCs under our experimental conditions. In partial agreement with our findings, others have reported that a small number of apoptotic cells are generated in response to GCs.(28)

We detected increased autophagic activity induced by Dex. Several standard approaches were adopted to determine autophagy based on recently published guidelines,(20) including fluorescent GFP-LC3 dots, MDC fluorescence, LC3 lipidation, and electron microscopic imaging, in addition to conventional acridine orange staining. The enhancement of autophagy also was validated in isolated primary osteocytes and osteocytes in bone from mice chronically treated with prednisolone. In addition, our gene microarray study showed the increased messenger level of several autophagy markers in the bone of the mice under GC treatment. The most dramatic elevation of autophagy markers was observed after 28 days of treatment. Interestingly, gene markers for proapoptosis, as well as osteoblast and osteocyte apoptosis, were not increased significantly until a later stage (56 days of chronic GC exposure).(8,29) Gene markers for autophagy were highly correlated with the gene markers for matrix proteolysis, including matrix metalloproteases (MMPs), caspases, and cathepsins.(29) The increased osteocytic autophagy and matrix proteolysis, in return, might induce the demineralization around the osteocytes that over time may weaken whole-bone strength.(8)

Autophagy was suppressed by 3-MA, which supports the hypothesis that Dex activates the autophagosomal pathway. More important, 3-MA, which blocks autophagy, actually enhanced the number of dead cells with GC treatment. In contrast to 3-MA, RU486 completely inhibited autophagy but partially rescued the reduction of viable cell numbers by Dex. One possible explanation is that RU486 is an antagonist of GC receptors that abolished any effects exerted by Dex, including autophagy induced by Dex. The rescue of cell number by RU486 is mainly due to the lack of Dex effect, which may not be directly related to the autophagy at all. 3-MA, on the other hand, may suppress autophagy activity directly. These data suggest that the increased autophagy is a likely self-responsive mechanism by osteocytes toward attenuating the effect of Dex on osteocytes. Autophagy, like a “double-edged sword,” has been reported to be involved in both cell protection and cell death.(11,12) It depends on the cell type and the level and duration of the stress. A unique feature of autophagy is that it is a protective mechanism against cell death probably initially or under short or moderate stress conditions. Our cell viability study showed that under our treatment regime, autophagic cells are very much alive and are likely under certain metabolic stress. Autophagy may be a mechanism whereby osteocytes can repair damaged organelles or cell membranes, such as in response to microcracks in pathologic overload. However, higher, persistent stress may generate a large accumulation of autophagosomes, leading to cell death. Indeed, apoptotic tunnel–positive osteocytes and osteoblasts are increased in vertebral cancellous bone of mice treated with 56 days of prednisolone.(3,30) Therefore, long-term treatment with GCs leads to cell death and, consequently, a reduction in bone strength and bone loss. Future studies are required to fully unravel the detailed mechanisms regulating the development and progression of autophagy in response to GC treatment.
